# Improvement in BMI z-score following adenotonsillectomy in adolescents aged 12–18 years: a retrospective cohort study

**DOI:** 10.1186/s12887-021-02634-y

**Published:** 2021-04-20

**Authors:** Keren Nathan, Galit Livnat, Liat Feraru, Giora Pillar

**Affiliations:** 1 Department of Pediatrics, Carmel Hospital and Technion Faculty of Medicine, Haifa, Israel; 2 Pediatric Pulmonary Unit & CF Center, Carmel Medical Center, Haifa, Israel; 3 Sleep Clinic, Carmel Hospital and Technion Faculty of Medicine, Haifa, Israel

**Keywords:** Obstructive sleep apnea (OSA), Adolescent, BMI z-score, Weight

## Abstract

**Background:**

Characteristics of obstructive sleep apnea (OSA) changes with age. Infants, toddlers and prepubertal children with OSA are usually underweight and may suffer from failure to thrive (FTT). Adenotonsillectomy (T&A) is the first line of treatment for OSA in childhood. In adults OSA is commonly associated with obesity and the metabolic syndrome. The change in body mass index (BMI) in adolescents with OSA following T&A was only sporadically studied. Thus, we peruse to examine the BMI z-score change following T&A in adolescents.

**Methods:**

Clalit Health Services is the largest health care organization in Israel with the largest patient registry (more than 50% of the population). Two hundred and forty two adolescents aged 12–18 who underwent T&A between 2006 and 2015 were identified in the Clalit registry and their characteristics including height and weight were retrieved. The BMI z-score of these adolescents at baseline (up to 3 months prior to T&A) and during the consecutive 3 years after T&A were analyzed and compared.

**Results:**

Changes in BMI Z-score were observed to all directions following T&A with overall small increase, not statistically significant (*P* = 0.26) from a median of 0.79 prior to T&A to a median of 0.835 after it. There was a minimal trend toward BMI z-score reduction in overweight children (*n* = 74) from 1.508 to 1.48 following T&A (*p* = NS), and in obese children (*n* = 33) from 2.288 to 2.000 (*P* = 0.06, 2 tailed). Interestingly thin individuals (*n* = 6) increased their BMI z-score following T&A from − 2.4 to − 0.59 (*p* = 0.046).

**Conclusions:**

Adolescents show variable changes in their BMI z-score following T&A. In this aspect their BMI z-score change is closer to the change seen in adults treated for OSA and not that of young children. The changes observed show a trend toward normalization of the BMI z-score such that overweight children tend to decrease their BMI z-score while thin individuals tend to increase it.

## Background

Obstructive sleep apnea (OSA) is a common childhood disorder with a prevalence of 1–6% in the pediatric population [1, 2] Clinically the syndrome is characterized by a disordered sleep with snoring and respiratory disturbances due to upper airway obstruction during sleep. For infants and toddlers untreated OSAS can lead to retardation of growth with a failure to thrive which is present in up to 56% of the children who are diagnosed with OSA [3, 4]. Risk factors for childhood OSA may be to some extent inheritance/genetics [5, 6] or obesity [7, 8]. Clearly adenotonsillar hypertrophy is the most common cause for OSA in children, and adenotonsillectomy (T&A) is considered the first line treatment. This is based both on the American Academy of Pediatrics (AAP) and the American Academy of Otolaryngology-Head and Neck Surgery (AAO-HNS) [9]. An association was found between OSA and cardiovascular impairment [10–12] as well as problems with learning and behavior in school aged children [13–15].

During infancy and early childhood, OSA frequently results in failure to thrive (FTT), in up to 50% of cases. Infants and toddlers under 4–6 years old with FTT seem to benefit from growth acceleration after conducting T&A with increase in BMI Z-score in these ages [16, 17]. During school years, weight gain was reported in children with OSA treated with T&A compared to children with OSA who were not treated at a 7–24 months follow up. The increase in BMI Z score was particular in children who were FTT, normal weight and overweight [18, 19]. In a twin study at the age of 3–12 years, T&A was associated with an increase of growth and IGF-1 levels compared to their twin none OSA sibling. An early surgical intervention made a significant growth recovery compared to the twin which had a lower BMI Z-score prior to surgery and had a greater increase in BMI Z-score following the surgery, compared with older children who had a greater BMI to begin with but the increase after T&A was minor [20, 21].

Obesity is a risk for residual and persistent OSA after T&A. Children with residual OSA showed greater weight gain after T&A compared to children without residual OSA [22–24]. For Adults tonsillectomy is not usually considered an isolated option to treat OSA as recommended by the American academy of sleep medicine [25]. However BMI is one of the outcome predictors to success for T&A in treating OSA similarly as with obese adolescents and children [23, 26]. BMI change in adults following treatment for OSA (for example with CPAP or oral appliance) is inconsistent, and studies had reported changes in all directions [27, 28]. Of note, recurrence of OSA was reported in adults after bariatric surgery even without concomitant weight increase [29]. T&A is the first line of treatment for childhood OSA but for children with mild to moderate OSA it is important to consider pharmacological options. Anti inflammatory treatment with intranasal corticosteroid (ICS) or montelukast or a combination have shown a beneficial effect (80% of participants) especially in the young and nonobese children [30]. A Cochrane review on the other hand suggested insufficient evidence for efficacy for intranasal ICS treatment and only a short term beneficial effect of montelukast [31].

Regardless of the treatment modality used, weight change following treatment was more intensively studied in adults and young children then in adolescents. The current study focusses on adenotonsillectomy (T&A). While in infants and young children T&A is associated with increase in BMI Z score and in adults, changes had been reported in all directions, there are very sparse data in this regard in adolescents. Therefore, the aim of the current study was to examine the BMI z-score change following T&A in adolescents, utilizing a large and well updated registry. We hypothesized that unlike young children, adolescents BMI change following T&A will be variable, similarly as seen in adults.

## Methods

Clalit Health Services is the largest health care clinics organization in Israel, insuring over 4.5 million people (more than 50% of the population). It maintains a very strict registry, which includes: patients records, physician visits and surgeries, several studies were published based on queries of this registry [32–34]. Each pediatrician (or ENT) visit is recorded in this registry, and pediatricians are encouraged to measure weight and height at least annually and record it in the registry. Of this registry, a retrospective investigation was preformed to include adolescents aged 12–18 years who underwent T&A in between 2006 and 2015. BMI z-score change from up to 3 months prior to T&A was compared to serially BMI measures every year in the 5 years following surgery (up to 2019). The years 2006–2015 were chosen to reflect a 10 years data period, allowing 3–4 years of follow up. The registry inquiry resulted in 242 adolescents who underwent T&A and had a BMI measurement (i.e. both height and weight) in the period of 3 months prior to the operation and at least one measurement in the 3 years following the surgery.

For each recognized case, the following data were pulled out from the registry: age, weight, height, gender, race, z score calculated from height and weight (at each timepoint). BMI z-score was categorized (based on the WHO) into 5 categories: Severe thinness (defined as SD < -3), thinness-underweight (SD − 2 to − 3), normal (SD − 2 to + 1), overweight (SD + 1 to + 2), obesity (SD > + 2).

statistical analysis was performed by using IBM statistics (SPSS) vs 24. The continuous variables were presented by mean, and standard deviation or Median & IQR, as appropriate. The categorical variables were presented in percentages. Differences in Z score between baseline and each time point, were assessed using paired t-test or Wilcoxon sign rank test, as appropriate (2 tails). Correlation between z scores and demographical and clinical characteristics were analyzed using Chi square test for the categorical variables and or Spearman correlation for the continuous variables. *P* < 0.05 was considered statistically significant.

The study was approved by the Israeli Clalit Helsinki community committee registered number 0222–16-com2. Participants did not have to sign a consent form. A waiver was obtained from the IRB committee. The data extracted from the registry did not include personal information such as patient’s names or identity numbers, each patient was assigned a serial number.

## Results

**Fig. 1:**
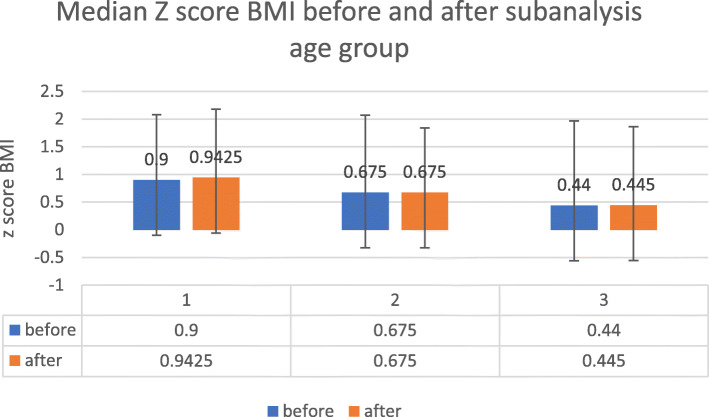
Median Z score in the 3 age groups 12-14 years (1), 14–16 years (2) and above 16 years (3)

Demographic data of the participants are listed in Table 1. Race and gender did not affect BMI Z-score outcome and therefore data are shown together for the whole cohort. Approximately 50% of the subjects in this cohort were in the normal range for BMI, z-score while 40% were overweight and obese. For the whole group (*n* = 242), there was a small trend of increase in BMI Z-score with a median of 0.79 prior to and 0.835 following T&A (*P* = 0.26). Categorization of the data based on age at the time of surgery is shown in Table 2 and Fig. 1**.** Data were divided into 3 age categories: 12–14 (132 subjects), 14–16 (60 subjects), and 16–18 (50 subjects) years. BMI z-score had changed to all directions. There was a trend to increase in BMI z-score in the younger age group from a median of 0.9 to 0.94 (*P* = 0.169), while in the other two age subgroups there were no changes in the median scores. BMI z-score category (defined by the WHO) changed in 25% of the children following the surgery, but the changes were in an inconsistent way and in all directions (Table 3). Of 242 participants 179 remained in their BMI z-score category (105 in normal BMI category, 53 overweight, and 21 obese). In normal weight category, 13 adolescents changed to higher BMI category while 9 changed to lower BMI category. There were even a few adolescents who had a change in two BMI categories with 4 obese subjects lowering their weight to normal range.

**Table 1:** General population characteristics

Variables	*N* = 242 (%)
Sex	
Female	143 (59.1)
Ethnic group	
Arabs	91 (37.6)
Jewish religious	6 (2.5)
General	145 (59.9)
Age category	
12–14 years	132 (54.3)
14–16 years	60 (24.7)
16–18 years	50 (20.6)
Z score category:	
Normal	127 (52.5)
Overweight	74 (30.6)
Obesity	33 (13.6)
Thinness	6 (2.5)
Severe Thinness	2 (0.8)

**Table 2:** BMI z-score data prior to and 3 years following T&A at 3 age group categories

Age Categories	Z-score BMI Before	Median Z score at 3 years	***P***-value
**12–14 years**	*N* = 132	N = 132	0.169
Mean	.7590	.7820	
Median	.9000	.9425	
Std. Deviation	1.17618	1.23738	
Percentiles			
25	−0.0575	0.1425	
50	0.9000	0.9425	
75	1.7800	1.8563	
**14–16 years**	*N* = 60	N = 60	0.306
Mean	0.3988	0.5395	
Median	0.6750	0.6750	
Std. Deviation	1.39603	1.16127	
Percentiles			
25	−0.5525	−0.2813	
50	0.6750	0.6750	
75	1.3650	1.3200	
**16–18 years**	*N* = 50	N = 50	0.173
Mean	0.4016	0.3999	
Median	0.4400	0.4450	
Std. Deviation	1.52683	1.41840	
Percentiles			
25	−0.8150	−0.6263	
50	0.4400	0.4450	
75	1.8200	1.6350

**Table 3:** Z-score categories prior to and 3 years following T&A-crosstabulation

	Median Z-score BMI at 3 years					Total
	Normal	Overweight	Obesity	Thinness	Severe Thinness	
Z score BMI before						
Normal	105	13	0	8	1	127
Overweight	10	53	11	0	0	74
Obesity	4	8	21	0	0	33
Thinness	4	1	0	0	1	6
Severe Thinness	1	0	0	1	0	2
Total	124	75	32	9	2	242

Table 4 details the BMI z-score change specifically for each BMI category. There were changes to all directions, resulting in very high standard deviations for the groups. About 30% of the children had changed their BMI category following the surgery, such that it tended toward the average (normalized). Overweight children (*n* = 74) tended to reduce their BMI z-score from 1.508 to 1.48 following T&A (p = none significant), and in obese children (*n* = 33) BMI z-score decreased from 2.288 to 2.000 accordingly (*P* = 0.06, 2 tailed). On the other hand, lean individuals (*n* = 6) increased their BMI z-score following T&A from − 2.4 to − 0.59 (*p* = 0.046), and in very thin individuals BMI Z-score increased dramatically from − 3.7 to − 1.1 (*p* = 0.18, *n* = 2 - underpowered).

**Table 4:** BMI z-score changes up to 3 years following T&A based on BMI z-score category prior to surgery

BMI z-score Categories	Z-score BMI Before	Median Z score at 3 years	***P***-value
**Normal**	*N* = 127	*N* = 127	0.140
Mean	−0.1665	−0.11	
Median	0.0500	0.14	
Std. Deviation	0.83631	1.024	
Percentiles			
25	−0.7600	−0.6500	
50	0.0500	0.1400	
75	0.5100	0.6300	
**Overweight**	N = 74	N = 74	0.798
Mean	1.5082	1.48	
Median	1.5250	1.53	
Std. Deviation	0.30918	0.504	
Percentiles			
25	1.5082	1.48	
50	1.5250	1.53	
75	0.30918	0.504	
**Obesity**	N = 33	N = 33	0.064
Mean	2.2882	2.00	
Median	2.2100	2.17	
Std. Deviation	0.24558	0.730	
Percentiles			
25	2.1150	1.8650	
50	2.2100	2.1700	
75	2.4000	2.4250	
**Thinness**	N = 6	N = 6	0.046
Mean	−2.4000	−0.59	
Median	−2.3350	−0.64	
Std. Deviation	.31875	1.707	
Percentiles			
25	−2.5950	−1.7000	
50	−2.3350	−0.6350	
75	−2.1775	0.7675	
**Severe thinness**	N = 2	N = 2	0.180
Mean	−3.6900	−1.11	
Median	−3.6900	−1.11	
Std. Deviation	0.35355	1.273	
Percentiles:			
25	−3.9400	−2.0100	
50	−3.6900	−1.1100	
75			

## Discussion

In this retrospective big data-based study performed on a large group of adolescents aged 12–18 years who underwent T&A for OSA. We observed that BMI z-score may change in any direction, with a tendency toward normalization of BMI z-score. Underweight and severe lean adolescents tended to increase their BMI z-score 3 years following the surgery, while overweight and obese individuals tended to reduce it. Age, gender and race had no effect on outcome and could not predict BMI z-score change following T&A. In the underweight category BMI Z-score increased significantly while in the severe thinness group there were only 2 individuals, but both showed a substantial BMI z-score increase following the surgery. Thus, it can be said that T&A worked in the direction of “normalization” of z-score.

It has been well documented that there is a strong link between OSA and the metabolic syndrome in adults which is multifactorial and is a consequence of visceral obesity and insulin resistance. There is a bidirectional feedforward association between them, with weight modification always being part of the treatment [35]. While infants with OSA are frequently underweight (usually suffer from failure to thrive), and adults with OSA are frequently overweight, in adolescents data are less clear, although in recent years adolescent OSA was reported to increase due to obesity [36, 37] .

In our study 60% of the adolescents were not obese or overweigh, which might partially explain why as a group there was a small trend of increase in BMI Z score following T&A. Interestingly, the two groups of overweight and obese accounting for approximately 40% of the participants tended to reduce their BMI Z score in the long term follow up after T&A. This is an encouraging and relatively novel finding for adolescents, indicating that OSA may contribute to obesity and thus OSA treatment may result in weight reduction as we have previously suggested in adults [38, 39]. In contrast, other studies exploring the association of T&A and weight reduction demonstrated an increase in BMI following T&A. Amin et al. demonstrated an increase in BMI after 1 year following T&A in school age children (7–13 years) especially for those who were obese at baseline. The velocity of the BMI increase after T&A was an independent risk factor for OSA recurrence [40]. Other studies including a systematic review as well showed similar results, but they all included wide age range from 0 to 18 years old with no stratification according to age subgroups and different outcome for obese and normal weight children [41, 42]. Potential mechanisms for weight reduction following T&A may include reduced appetite (which is known to be high in sleepy individuals due to Ghrelin secretion), increase in physical activity (which is a direct result from reduction of sleepiness), and improvement in insulin sensitivity. Other components of the metabolic syndrome, lipid profile and insulin resistance in obese children and adolescents have been shown to improve with the resolution of OSA after T&A [28, 43, 44]. Our data in this study supports the notion of having two OSA phenotype that change around puberty, from lymphadenoid hypertrophy to an OSA related more to obesity [45]. The unpredicted changes observed to all directions in our study resembles findings from juvenile rat studies and clinical trials, in which the removal of airway obstruction was not able to restore the dysregulated hormonal axes [46] and other lifestyle behaviors (high caloric intake, physical activity) had a strong influence [47, 48]. Seven weeks following obstruction removal in juvenile rats showed that these animals still had high ghrelin levels and consumed more food, yet exhibited growth retardation due to deregulation of GH homeostasis [46]. Although the participants in the current study were adolescents (not exactly parallel to juvenile rats), increased ghrelin levels and food consumption, along with dysregulation of GH release, may explain substantial increase in BMI Z score as was observed in our underweight and severe lean participants, although such potential mechanisms will require a future study.

In a study aimed to examine anatomical parameters in adolescents using MRI, obese adolescents with OSAS had increased adenotonsillar tissue compared with obese and lean control subjects (total of 137 subjects) without OSAS. Lymphoid tissue, rather than other soft tissue components, was the primary structural abnormality in obese adolescents. This finding supports adenotonsillectomy to be considered as treatment for OSAS in these obese adolescents, as opposed to adults in whom T&A is commonly insufficient, requiring treatment with CPAP or weight reduction [49]. In another study, in obese children and adolescents aged 7–18 years, OSA improved with weight reduction in the same manner as obese adults are responding to weight reduction [50]. Thus, our study indicates that T&A may improve weight, suggesting that OSA itself contributes to the weight increase process in adolescents.

On the other hand, very lean (underweight and severe underweight) adolescents responded with BMI z-score increase following T&A. The mechanism for this is unclear. In infants OSA is associated with FTT with suggested mechanisms of reduced calorie intake and increased energy expenditure due to work of breathing. We did not assess food intake or work of breathing in our retrospective study and cannot confirm or rule out these mechanisms in our adolescents. However, it is important to point out that of the 8 cases in these categories, 6 gained weight and moved up to normal BMI category, and only 1 individual became overweight the remaining 8th individual reduced weight and changed from thinness to severe thinness category (Table 3). Obviously, our study suffers from being underpowered for underweight adolescents (*n* = 8), yet due to the dramatic weight gain these changes were statistically significant. Further studies are required to better understand this (small) subgroup of underweight adolescents with OSA, and the effect of T&A on them.

The strengths of our study are the large number of participants, being a real “field” study indicating changes observed in “real life” with no research intervention, and the relatively large follow up period (3 years for all, in some up to 5 years). On the other hand, there are several limitations in our study. First, this is a retrospective data base study. However, we believe that the well-kept registry of Clalit together with the large number of participants still makes the results of this study valid and representative. Actually, the fact that this is a “field study” report and not prospective weight-change follow-up study makes it somewhat more representative of the “real” clinical world. Second, PSG numerical results (i.e. apnea hypopnea index, oxygen saturation, sleep time etc.) were not available from the Clalit registry. We only had the diagnosis code for OSA. Thus, we cannot provide data regarding the effect of T&A on OSA in our study, or the correlation between change in OSA and change in BMI z-score. We can only make a logical guess that at least in those with substantial weight reduction following T&A OSA was improved but again we cannot support it by data in our study. However, it is supported by some data from previous studies. Finally, the data represents only the insurers of Clalit, thus cannot be automatically generalized for the whole Israeli population. However, we believe that it is quiet representative and are unaware of any specific weight or OSA related data and preference of other medical insurance. Thus, we believe these results are representative and generalizable.

## Conclusion

Despite the above-mentioned limitations, we believe our study results are valid and representative. Adolescents following T&A show variable changes in BMI z-score, with a tendency toward normalization of it. In the subgroups of underweight T&A resulted in BMI z-score increase, while in overweight and obese adolescents, it resulted in a tendency toward BMI z-score reduction. In these regards, the observations resemble the changes seen in adults and not in young children. Further studies are needed in this age group to characterize adolescent’s OSAS features and BMI change following T&A, and the correlation between them.

## Data Availability

The data that support the findings of this study are available from Clalit data committee but restrictions apply to the availability of these data, which were used under license for the current study, and so are not publicly available. Data are however available from the authors upon reasonable request and with permission of Clalit data committee.

## Abbreviations

BMI: Body mass index
CPAP: Continues positive airway pressure
FTT: Failure to thrive
OSAS: Obstructive sleep apnea syndrome
T&A: Tonsillectomy and adenoidectomy
